# Posterior reversible encephalopathy syndrome while receiving irinotecan with fluorouracil and folinic acid for metastatic gastric cancer

**DOI:** 10.1259/bjrcr.20170033

**Published:** 2017-11-17

**Authors:** Omar K Abughanimeh, Ayman H Qasrawi, Mohammad Y Tahboub, Mouhanna K Abu Ghanimeh

**Affiliations:** ^1^Department of Internal medicine, University of Missouri-Kansas City School of Medicine - Graduate Medical Education, Kansas City, MO, USA; ^2^Department of Internal medicine-Division of gastroenterology, Henry Ford Health System, Gastroenterology, Detroit, MI, USA

## Abstract

Posterior reversible encephalopathy syndrome (PRES) is a clinical-radiographic syndrome with seizures, headache, altered mental status and visual disturbances. It is typically associated with posterior cerebral white matter oedema on neuroimaging. There is an increasing number of cases of PRES reported with different chemotherapeutic protocols. However, PRES is rarely reported in association with irinotecan, fluorouracil and folinic acid (FOLFIRI). We report a 28-year-old female patient with a history of Stage IV gastric cancer who presented with abdominal pain and recurrent vomiting that was thought to be related to a partial intestinal obstruction secondary to peritoneal metastasis. Eventually, she was treated with FOLFIRI. A few hours after initiation of the fluorouracil infusion in the second cycle, she developed a tonic-clonic seizure. MRI of the brain showed multiple bilateral *T*_2_ and flair hyperintense cortical and subcortical lesions suggestive of PRES. Other causes of PRES were excluded, as well as brain metastasis. Unfortunately, the patient developed septic shock and died a few days after her presentation.

## Background

Posterior reversible encephalopathy syndrome (PRES) is a distinct clinical-radiographic syndrome that was first described by Hinchey et al.^1^ PRES presents with seizures, headache, altered mental status or visual disturbances, associated with posterior cerebral white matter oedema on neuroimaging.^1,2^ Even though PRES is a benign and reversible condition,^1,3^ it can also can be fatal or lead to permanent neurological disability.^4^ Thus, an early diagnosis of PRES is crucial to prevent complications.^5^ Chemotherapeutic agents are uncommonly recognized as risk factors or causative agents for PRES.^2^ A toxic damage to the vascular endothelium, blood brain barrier (BBB) dysfunction by cytotoxic effect and a possible modulation of inflammatory cascades can lead to increased BBB permeability with resulting oedema.^5–7^ Treatment of PRES is supportive by controlling blood pressure and stopping the inciting factor.^2,7^ In this case report, a 28-year-old female patient with a history of Stage IV gastric cancer developed PRES while she was receiving chemotherapy. Other potential causes of her presentation as well as other causes of PRES were excluded. Unfortunately, she developed septic shock and died a few days afterward.

## Case summary

### Clinical presentation

Our patient was a 28-year-old female individual with a past medical history of benign essential hypertension and Stage IV gastric cancer with peritoneal metastasis, for which she had completed five cycles of docetaxel, cisplatin and 5-flurouracil (DCF). CT scan of the abdomen and pelvis 3 weeks after the last cycle of DCF showed disease progression and worsening peritoneal metastasis.

Two weeks later, she presented to the emergency room with abdominal pain and recurrent vomiting for the last few days. On evaluation, she was in distress. Her temperature was 37.6°C, her heart rate was 106 beats min^–1^ and her blood pressure was 139/87 mmHg. The abdomen was slightly distended with mild tenderness to palpation in the epigastric area. No rebound tenderness, guarding or rigidity were noted. CT scan of the abdomen and pelvis with contrast showed a focal dilatation of proximal jejunal loops suggestive of partial intestinal obstruction. The patient was admitted for medical management of the intestinal obstruction and surgical consultation.

The patient was started on intravenous hydration and was kept nil per os (NPO). The serum amylase and lipase levels were normal. The surgery team decided to continue with conservative treatment owing to a high surgical risk and peritoneal metastasis. After a few days, the pain and vomiting improved.

The tumour board committee thought that the jejunal obstruction was due to disease progression. They decided to start irinotecan intravenously (i.v.) 180 mg m^–2^, folinic acid i.v. 400 mg m^–2^ and fluorouracil i.v. 400 mg m^–2^ as a bolus dose, followed by 2400 mg m^–2^ i.v. infusion over 46 h (FOLFIRI chemotherapy regimen).

The vomiting recurred after the initiation of chemotherapy and was worse than before, which warranted keeping her as an inpatient. Multiple antiemetics were used to control her vomiting, including metoclopramide, ondansetron, lorazepam and eventually an octreotide i.v. infusion.

The patient’s vomiting was fairly controlled with the octreotide infusion and the above antiemetic regimen. The second cycle of FOLFIRI was started 14 days after the first cycle with the same doses. 4 hours after initiation of the fluorouracil infusion, she developed a tonic-clonic seizure for 2 min, which was controlled with 5 mg i.v. of diazepam.

The patient was haemodynamically stable after the seizure with mild hypertension (157/87 mmHg). Her neurological examination was non-focal. The fluorouracil was stopped and phenytoin i.v. 1000 mg was given.

## Investigation

**Figure 1.**
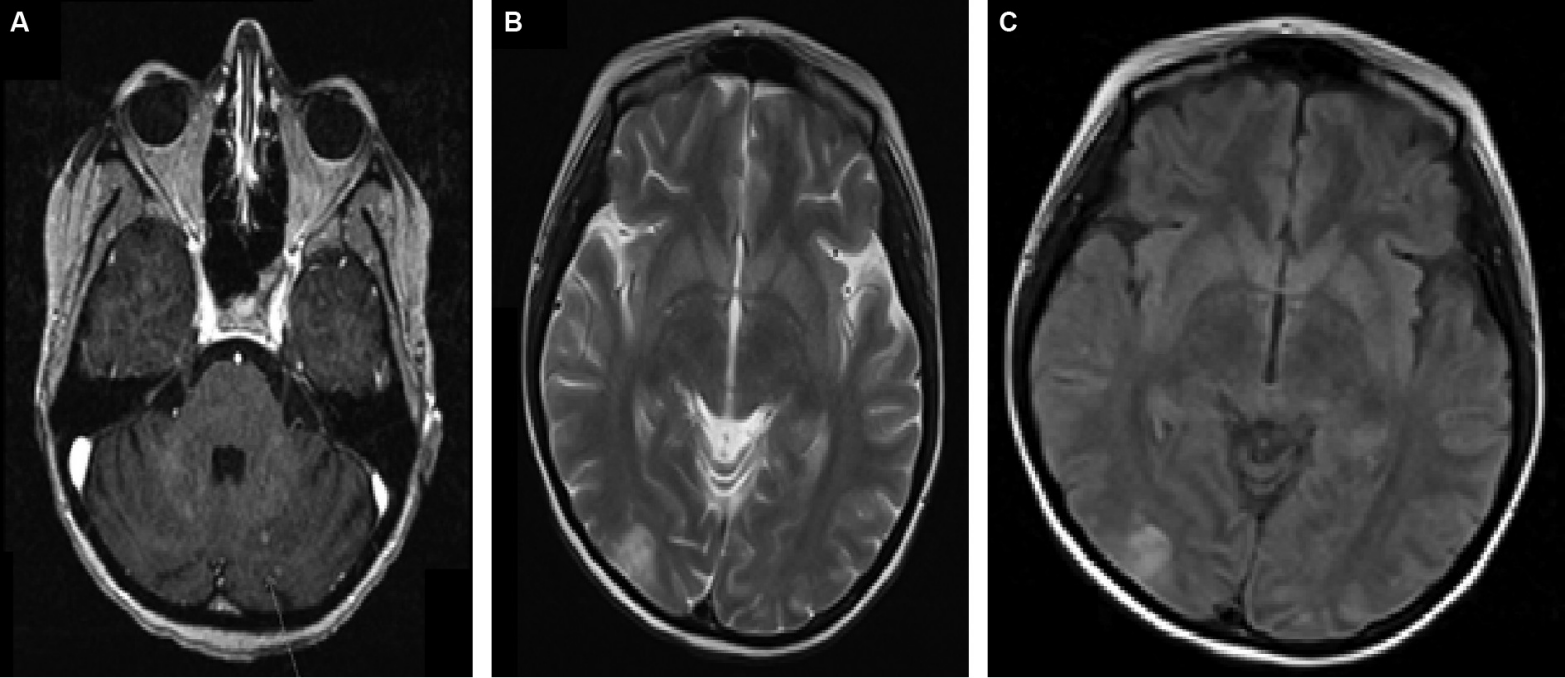
Brain MRI with contrast. (a) *T*_1_ sequence, (b) *T*_2_ sequence, (c) *T*_2_-flair sequence. These sequences show multiple bilateral *T*_2_ and flair hyperintense cortical and subcortical lesions in the parietal and occipital lobes, as well as in the cerebellar hemispheres. Some lesions showed minimal enhancement. No significant surrounding oedema, intracranial haemorrhage, space-occupying lesions, hydrocephalus or midline shift are seen.

A brain MRI with contrast (Figure 1) was done. *T*_1_ (Figure 1a),* T*_2_ (Figure 1b) and *T*_2_-flair (Figure 1c) showed multiple bilateral *T*_2_ and flair hyperintense cortical and subcortical lesions in the parietal and occipital lobes, as well as in the cerebellar hemispheres. Some lesions showed minimal enhancement. No restriction was noted on diffusion-weighted images. There was no significant surrounding oedema, intracranial haemorrhage, space-occupying lesions, hydrocephalus or midline shift. Brain metastasis was less likely given the acuity of onset, the absence of brain metastasis on previous brain MRI, the absence of enhancing lesions at the gray-white matter junction and the absence of distortion of the brain architecture. Her electrolyte levels were unremarkable. An echocardiogram with bubble study (was done 3 days after the seizure) did not show any vegetations or intracardiac shunts. These findings were consistent with PRES.

### Follow-up

Unfortunately, the patient developed *Klebsiella* bacteremia and septic shock 2 days after the seizure and was transferred to the intensive care unit. She deteriorated despite all efforts, and died after 7 days.

## Discussion

PRES is a clinical-radiographic syndrome of seizures, headache, altered mental status and visual disturbances associated with posterior cerebral white matter oedema on neuroimaging. PRES was first described by Hinchey et al.^1^ Interestingly, this syndrome is not always reversible, and the cerebral oedema can involve any part of the brain. Therefore, the name is not completely satisfactory.

The exact incidence of PRES is unknown. It affects all age groups and is more common in female individuals.^2–4^ Tam et al^5^ specified particular risk factors for PRES, including significant fluid overload (>10% of baseline weight), increased blood pressure (>25% of baseline) and creatinine > 1.8 mg dl^–1^. Any of these risk factors should promote the use of early neuroimaging to evaluate any unexplained neurological change.^5^

Chemotherapy-associated PRES is uncommon, even though there is an increasing number of reported cases.^2,5–8^ How et al ^2^ included 70 cases in their systematic review on chemotherapy-associated PRES. Most of the cases presented within the first week of chemotherapy. The most commonly encountered chemotherapeutic agents were platinum-based agents (cisplatin, carboplatin and oxaliplatin—30 cases), daunorubicin (24 cases) and vinca alkaloids (vincristine, vinorelbine, vinflunine, vinblastine and vindesine—21 cases). There were 13 cases associated with 5-fluorouracil and 1 case associated with irinotecan. None of the cases were associated with FOLFIRI. To the best of our knowledge, this case is the second reported case of PRES after FOLFIRI treatment alone. Additionally, our case is the first case that includes gastric cancer [Table 1].

**Table 1.** Reported cases of PRES with FOLFIRI chemotherapy

Case/primary malignancy	Patient’s age/gender	Chemotherapy regimen	Presenting symptom	Onset (days after chemotherapy)	Involved brain areas on neuroimaging
Plavetić et al^6^/ Stage IV colorectal cancer	45-year-old/ female	FOLFIRI	Headache and tonic-clonic seizures	5 days	The occipital and posterior parietal lobes
Allen et al^7^/ Stage IV colorectal cancer	52-year-old/ male	FOLFIRI/bevacizumab	Headache and bilateral cortical blindness	14 days	The occipital and posterior parietal lobes
Abughanimeh et al (our* case)*/Stage IV gastric cancer	28-year-old/ female	FOLFIRI	Seizure	First day (2 h after the fluorouracil infusion)	Cortical and subcortical lesions in the parietal and occipital lobes. Cerebellar hemispheres

There is no clear explanation for the pathophysiology of chemotherapy-associated PRES. Toxic damage to the vascular endothelium and BBB dysfunction due to cytotoxic medications could explain part of it. Additionally, cytotoxic medications can increase tumour cell recognition, which stimulates an inflammatory cascade that can lead to increased BBB permeability and axonal swelling that leads to vasogenic oedema.^5–7^

In general, the most common neuroimaging finding in PRES is oedema of the white matter in the posterior portions of the cerebral hemispheres, especially in the bilateral parieto–occipital regions.^1^ However, any part of the brain could be involved, including the brain stem, cerebellum or basal ganglia.^1^ In chemotherapy-associated PRES, involvement of the temporal and frontal lobes is possible as well.^2^ It has been noted that involvement of the occipital lobes increases the risk of having a seizure.^9^

Finally, PRES is usually a benign condition. Many cases seem to be fully reversible within days to weeks after removal of the inciting factor and with good blood pressure control.^1,3^ However, PRES can also be fatal or lead to permanent neurological disability.^4^

## Conclusions

Chemotherapy-associated PRES is uncommon, but has been reported. It shares many clinical and radiological characteristics with other cases of PRES. Early diagnosis of PRES as well as recognition of risk factors is important to prevent complications.

## Learning points

1. PRES is a clinical-radiographic syndrome with a variety of clinical features and characteristics on neuroimaging.
2. The most common neuroimaging finding with PRES is oedema of the white matter in the posterior portions of the cerebral hemispheres, especially in the bilateral parieto–occipital lobes.
3. Risk factors for PRES include significant fluid overload, worsening hypertension and impaired kidney function.
4. Chemotherapy-associated PRES is uncommon but has been reported.

## Consent

Written informed consent for the case to be published (including images, case history and data) was obtained from the patient(s) for publication of this case report, including accompanying images.
